# Biomarkers in Progressive Fibrosing Interstitial Lung Disease: Optimizing Diagnosis, Prognosis, and Treatment Response

**DOI:** 10.3389/fmed.2021.680997

**Published:** 2021-05-10

**Authors:** Willis S. Bowman, Gabrielle A. Echt, Justin M. Oldham

**Affiliations:** Division of Pulmonary, Critical Care, and Sleep Medicine, University of California, Davis, Davis, CA, United States

**Keywords:** interstitial lung disease, biomarker, progressive fibrosing ILD, idiopathic pulmonary fibrosis, hypersensitivity pneumonitis, connective tissue disease-associated interstitial lung disease

## Abstract

Interstitial lung disease (ILD) comprises a heterogenous group of diffuse lung disorders that commonly result in irreversible pulmonary fibrosis. While idiopathic pulmonary fibrosis (IPF) is the prototypical progressive fibrosing ILD (PF-ILD), a high proportion of patients with other ILD subtypes develop a PF-ILD phenotype. Evidence exists for shared pathobiology leading to progressive fibrosis, suggesting that biomarkers of disease activity may prove informative across the wide spectrum of ILDs. Biomarker investigation to date has identified a number of molecular markers that predict relevant ILD endpoints, including disease presence, prognosis, and/or treatment response. In this review, we provide an overview of potentially informative biomarkers in patients with ILD, including those suggestive of a PF-ILD phenotype. We highlight the recent genomic, transcriptomic, and proteomic investigations that identified these biomarkers and discuss the body compartments in which they are found, including the peripheral blood, airway, and lung parenchyma. Finally, we identify critical gaps in knowledge within the field of ILD biomarker research and propose steps to advance the field toward biomarker implementation.

## Introduction

Interstitial lung disease (ILD) comprises a heterogeneous group of diffuse parenchymal lung processes characterized by variable degrees of inflammation and fibrosis. While etiology varies by ILD subtype, studies suggest that pathobiology leading to pulmonary fibrosis may be shared across subtypes (1, 2). Idiopathic pulmonary fibrosis (IPF) is among the most common forms of fibrotic-predominant ILD and is characterized by progressive parenchymal fibrosis leading to clinical deterioration and high mortality (3). Other common forms of ILD, including connective tissue-associated ILD (CTD-ILD), chronic hypersensitivity pneumonitis (CHP), and unclassifiable ILD (uILD) manifest variable patterns of inflammation and fibrosis and can approximate the natural history of IPF when progressive (2, 4). Immunosuppressive therapy is thought to provide benefit for patients with inflammatory ILD (5, 6), but was found to cause harm to patients with IPF (7), underscoring the importance of an accurate diagnosis. Patients with IPF are now treated with antifibrotic therapy, which slowed disease progression in several randomized controlled trials (8–11). More recently, anti-fibrotic therapy was shown to provide similar benefit in patients with CTD-ILD, CHP, uILD, and others with progressive fibrosing ILD (PF-ILD) phenotype (12, 13).

Despite advances in the treatment of PF-ILD, predicting a progressive phenotype remains difficult. Biomarkers have advanced our understanding of IPF and are now beginning to inform diagnosis, prognosis, and treatment in patients with ILD, including those with PF-ILD. In this review we provide an overview of the current state of ILD biomarker research, including relevant biomarkers to help discriminate ILDs, prognosticate disease course, and assess treatment response. Finally, we highlight unmet needs in ILD biomarker research and discuss strategies for biomarker implementation.

## The PF-ILD Phenotype

A number of clinical criteria for identifying a PF-ILD phenotype have been proposed. While few have yet been validated, they generally utilize longitudinal measures of progressive disease, including lung function decline, worsening respiratory symptoms, and increasing extent of radiologic fibrosis (12–15). Defining PF-ILD by presence of these features stems largely from their known association with worse outcomes in ILD. Longitudinal decline in forced vital capacity (FVC) is perhaps the best near-term predictor of subsequent mortality. While a categorical FVC decline of ≥10% has been associated with an increased mortality risk in patients with IPF (16–19), CHP (20–22), and CTD-ILD (23), even modest FVC decline of≥5% decline has been linked to worse outcome in IPF (16). Baseline extent of fibrosis on high-resolution computed tomography (HRCT) has been linked to prognosis in different ILD subtypes (24, 25), even in the absence of FVC decline (26), and suggests that longitudinal increase in fibrosis extent is a good measure of progressive ILD (25, 27, 28). Symptomatic worsening, either alone or in combination with FVC decline or fibrotic progression on HRCT also suggests ILD worsening, but has yet been to validated as a reliable predictor of outcome.

## Biomarker Acquisition

A biomarker is defined as an indicator of normal biological processes, pathogenic processes, or responses to an exposure or intervention, including therapeutic interventions (29). Sources of biomarkers that may inform diagnosis, outcomes, and treatment response in ILD include the peripheral blood, airway, and lung parenchyma. Peripheral blood is easily obtained, and acquisition requires little training beyond phlebotomy. While airway biomarkers may be obtained non-invasively via exhaled breath, most studies to date have employed bronchoscopy to obtain these biomarkers via bronchoalveolar lavage. Obtaining parenchymal biomarkers requires increasingly invasive bronchoscopic approaches depending on specimen size or by surgical lung biopsy, usually by way of video-assisted thoracoscopic surgery. Novel functional and quantitative imaging techniques are also emerging as non-invasive parenchymal biomarkers (30–32).

While peripheral blood is easy to obtain in cross-section and serially over time, biomarkers from this compartment may not reflect pathobiologic processes in the lung. Conversely, while those obtained from the airway or lung parenchyma may better reflect the parenchymal pathobiology, invasive techniques are required generally for acquisition and limit the widespread use of these modalities along with serial biospecimen acquisition. Radiologic biomarkers do allow for serial acquisition, but radiation exposure remains a consideration, especially among younger individuals with ILD. Biomarkers within each compartment of interest can be further classified according to the endpoints with which they are associated, including disease discrimination, prognosis, and treatment response. Below we highlight key biomarkers within each compartment and the endpoints they help predict in patients with ILD.

## Peripheral Blood Biomarkers

### Diagnostic

A large number of plasma and serum protein biomarkers discriminate ILD from healthy controls, ILD within in a disease state, and between ILD subtypes (Table 1) (33–69, 71–104). Despite the considerable work performed in this arena, test performance for individual biomarkers has generally been insufficient to justify their incorporation into clinical practice. Recent investigations have demonstrated increased potential when modeling plasma biomarkers in aggregate. Doyle et al. demonstrated matrix metalloproteinase 7 (MMP-7), pulmonary and activation-related chemokine (PARC), and surfactant-protein D (SP-D) effectively predicted ILD in patients with rheumatoid arthritis (RA) when modeled with clinical characteristics (84). White and colleagues then employed a similar approach, using plasma MMP-7, SP-D, and osteopontin concentration to derive and validate a protein prediction score to distinguish IPF from healthy controls and most alternative ILDs (85). One group for which this scoring system performed poorly was RA-ILD, which shares morphologic features with IPF. Recently, Sanders et al. showed that biomarkers hold promise for identifying early ILD, termed interstitial lung abnormalities (ILAs). These authors found that GDF15 and four other plasma biomarkers were increased in community dwelling adults with ILAs (48), with GDF15 validated in an independent cohort. These findings suggest that plasma biomarkers may eventually allow for non-invasive ILD screening (105).

**Table 1:** Diagnostic ILD plasma/serum biomarkers.

**Biomarker**	**ILD subtype**	**Discriminates vs. non-ILD control**	**Discriminates vs. Other ILD subtype**	**References**
AGE/sRAGE	IPF	+	+ (idiopathic NSIP)	(33)
	CHP	+	+ (idiopathic NSIP)	(33)
Apolipoproteins	IPF		+ (CHP)	(34)
CA 15-3	RA-ILD	+		(35)
CA 19-9	RA-ILD	+		(35)
CA-125	RA-ILD	+		(35)
Calgranulin (S100)	IPF	+		(36)
CC16	IPF	+	+ (CHP, CTD-ILD)	(36, 37)
	SSc-ILD	+		(38)
CCL-2	ILD	+		(39)
	SSc-ILD	+		(40)
CCL-3	SSc-ILD	+		(40)
CCL-11	IPF	+		(41)
CCL-15	CHP	+		(42)
CCL-18	IPF	+		(43)
	SSc-ILD	+		(44, 45)
CD59	IPF	+		(46)
CRP	SSc-ILD	+		(47)
	ILA	+		(48)
CX3CL1	SSc-ILD	+		(49)
CXCL1	IPAF	+	+ (IIP)	(50)
CXCL4	SSc-ILD	+		(51)
CXCL9	CHP	+		(52)
CXCL10	RA-ILD	+		(53)
	SSc-ILD	+		(54)
CXCL13	IPF	+		(55)
E-selectin	SSc-ILD	+		(56, 57)
FAS	IPF	+		(41)
GDF-15	SSc-ILD	+		(58–60)
	ILA	+		(48)
	IPF	+		(61)
ICAM-1	SSc-ILD	+		(62)
	IPF	+		(43)
IGFBP-1/2	IPF	+		(63)
IL-4	SSc-ILD	+		(64)
IL-6	SSc-ILD	+		(65)
IL-8	IPF	+		(66)
IL-10	IPF	+		(66)
IL-12	IPF	+		(66)
	IIM-ILD	+		(67)
IL-12B	IPF	+		(41)
IL-33	SSc-ILD	+		(68)
IL-35	SSc-ILD	+		(69)
KL-6	RA-ILD	+		(70)
	ILD	+		(71)
	CHP	±		(52, 72, 73)
	IPF	+		(39, 74, 75)
	SSc-ILD	+		(44, 56, 74, 76–79)
	CTD-ILD	+		(80)
MMP-degraded Proteins	IPF	+		(81, 82)
MMP-1	IPF	+	+ (CHP, sarcoid)	(41)
	SSc-ILD	–		(83)
MMP-3	IPF	+		(41)
MMP-7	RA-ILD	+		(53, 84)
	IPF	+	+ (CHP, Sarcoid, other IIPs)	(41, 74, 85)
	SSc-ILD	+		(74, 86)
MMP-8	IPF	+		(41)
MMP-9	IPF	+		(41)
MMP-12	SSc-ILD	+		(87)
Napsin A	IPF	+		(75)
Nucleosomes	IPF	+		(88)
PARC	RA-ILD	+		(84)
Periostin	CHP	+		(89)
	IPF	+		(90)
SP-A	IPF	+	+ (NSIP)	(36, 75, 91–93)
	SSc-ILD	+		(94)
SP-D	IPF	+	+ (other IIPs)	(36, 43, 74, 75, 85, 95)
	SSc-ILD	+		(44, 74, 76, 77, 94, 96, 97)
	RA-ILD	+		(84)
TFF-3	IPF	+		(36)
TIMP-1	SSc-ILD	±		(83, 98)
TNFR	IPF	+		(41)
Wnt5a	RA-ILD	+		(99)
YKL-40	CHP	+		(100)
	IPF	+		(101, 102)
	SSc-ILD	+		(103)
	IIM-ILD	+		(104)

Genomic investigation of DNA acquired from peripheral blood has identified several common gene variants associated with ILD. The variant with strongest effect is a polymorphism in the promoter region of *MUC5B*, which encodes a mucin producing gene critical for airway host defense (106). The presence of this variant increases the risk of developing IPF by 5-fold (107–109) and was recently shown to increase the risk of developing rheumatoid arthritis (RA)-associated ILD, especially with concurrent usual interstitial pneumonia (UIP) pattern (110, 111). Interestingly, the *MUC5B* promoter variant does not appear to increase risk for ILD due to systemic sclerosis or anti-synthetase syndrome (112, 113). A number of other variants have been linked to IPF susceptibility but with smaller effect association (109, 114, 115). While these studies were informative, it is unlikely that common gene variants, including the *MUC5B* promoter variant, will allow for cost-effective ILD screening, as such variants are found in a large minority of unaffected individuals.

### Prognostic

In addition to discriminating ILD presence, many of the biomarkers described above have also been linked to prognosis, including survival, near-term lung function decline and ILD exacerbation (Table 2) (116–153). Plasma biomarkers most commonly linked to differential survival include chemokine (C-C motif) ligand 18 (CCL18) (44, 126, 127, 129, 130), Krebs von den Lungen 6 (KL-6) (131, 133, 138, 142), chitinase-3-like protein 1 (YKL-40) (101, 104, 117), cancer antigen 125 (CA-125) (117–119), and MMP-7, (117, 121, 145, 153) which have been shown to predict this endpoint across diverse forms of ILD. Our group recently showed that several of these biomarkers predicted differential survival in both antifibrotic treated and untreated patients with IPF, but at higher categorical thresholds in anti-fibrotic treated patients (117).

**Table 2:** Prognostic ILD plasma/serum biomarkers.

		**Outcome predicted**			
**Biomarker**	**ILD subtype**	**Lung function decline**	**Survival**	**Exacerbation**	**References**
AGE/sRAGE	IPF		+		(33)
Anti-MDA-5 Ab	IIM-ILD		+		(116)
CA-125	IPF	+	+		(117, 118)
	CTD-ILD		+		(119)
	CHP		+		(119)
	uILD		+		(119)
CA19-9	IPF	+	+		(118)
Calgranulin (S100)	IPF	+	+		(120, 121)
CCL-2	SSc-ILD	+	+		(122, 123)
	IIM-ILD		+		(124)
CCL17	CHP	+			(52)
CCL-18	IPF	+		–	(125–127)
	SSc-ILD	±	+		(44, 128–131)
CD59	IPF		+		(46)
CRP	SSc-ILD	+	+		(47)
CX3CL1	SSc-ILD	+			(49)
CXCL4	SSc-ILD	+			(132)
CXCL9	CHP	+			(52)
CXCL10	IIM-ILD		+		(124)
CXCL13	CTD-ILD		+		(119)
	CHP		+		(119)
	uILD		+		(119)
	IPF	+	+		(55, 119, 133, 134)
GDF-15	IPF		+		(61)
ICAM-1	SSc-ILD	+			(62)
	IPF	+	+		(121)
IgA	IPF		+	+	(135)
IL-6	SSc-ILD	+	+		(136)
	IIM-ILD		+		(137)
IL-10	SSc-ILD	+	-		(122)
KL-6	IPF	+	+	+	(125, 133, 138–141)
	SSc-ILD	+	+		(79, 96, 131, 142)
LOXL-2	IPF		+		(143)
MMP-degraded Proteins	IPF	+	+		(81, 82)
MMP-3	CTD-ILD		+		(144)
	IPF		+		(134)
MMP-7	CTD-ILD		+		(119)
	CHP		+		(119)
	uILD		+		(119)
	IPF	+	+		(117, 121, 145)
	IIM-ILD		+		(145)
MMP-9	CTD-ILD		+		(144)
MMP-10	IPF	+	+		(146)
Neopterin	IIM-ILD		+		(147)
Osteopontin	IPF		+		(117)
	CTD-ILD		+		(144)
Periostin	IPF	+	+		(90, 148, 149)
	CHP		+	+	(89)
SP-A	IPF	+	+		(145)
SP-D	IPF	+	+		(36, 74, 118)
	SSc-ILD	+			(44, 74)
VCAM-1	CTD-ILD		+		(119)
	CHP		+		(119)
	uILD		+		(119)
	IPF	+	+		(117, 121)
	IIM-ILD		+		(150)
VEGF	IPF	+			(151)
VEGF A165b	IPF	+			(152)
YKL-40	IPF		+		(101, 117)
	CHP			+	(100)
	SSc-ILD		+/-		(103)
	IIM-ILD		+		(104)

While most biomarkers are measured in cross-section, Maher and colleagues showed longitudinal change in plasma CA-125 concentration to predict subsequent mortality in a prospectively recruited IPF cohort (118). Neoepitopes of protein fragments created during extracellular matrix turnover may also prove to be valuable biomarkers. Several have been shown to predict subsequent IPF survival when modeled in cross-section and over time (81, 82). Additional novel plasma biomarkers of ILD survival are expected in the coming years as high-throughput proteomic platforms are increasingly utilized (154).

Other blood-based biomarkers also hold potential. Monocyte count, obtained as part of a complete blood count, was recently shown to predict increased mortality across multiple IPF cohorts (155). Kreuter et al. also showed increasing monocyte count to predict near-term hospitalization and IPF progression, as measured by death, decreasing walk distance or ≥10% categorical decline in FVC (156). Because FVC decline is among the earliest objective indicators of a progressive phenotype and change in FVC among the most commonly utilized primary endpoint in clinical trials, biomarkers predictive of FVC decline are of particular importance for identifying early progression and enriching clinical trial cohorts. CCL18 (126), MMP7 (157), SP-D (74, 158), and interleukin-6 (IL-6) (136) have each demonstrated potential in predicting near-term FVC decline in patients with ILD, though none with sufficient risk explanation to justify clinical implementation to date.

Genomic biomarkers have also been linked to differential ILD survival. The *MUC5B* promoter SNP, despite increasing IPF risk (108), was paradoxically associated with reduced mortality in IPF (159). This observation may be been driven by index event bias (160), leaving it unclear what role *MUC5B* plays in ILD progression. The same polymorphism has also been associated with increased mortality risk in patients with CHP (161). Short leukocyte telomere length has been shown to predict survival across numerous ILD cohorts and subtypes (161–163) and patients with rare mutations in telomerase encoding genes, including *TERT, TERC, PARN*, and *RTEL1* were shown to display survival similar to IPF irrespective of the clinical ILD diagnosis (164). Recent studies have also suggested circulating mitochondrial DNA may be a relevant biomarker for predicting IPF outcomes (165, 166). Transcriptomic biomarkers also appear informative, as a 52-gene signature has been shown to reliably predict IPF survival across numerous cohorts in the US and Europe (167, 168).

### Treatment Response

Compared to the biomarkers of diagnostic and prognostic utility in ILD, there is less evidence supporting the role of biomarkers to predict or monitor treatment response in patients with ILD. Serial measurements of KL-6, SP-D, IL-6, CXCL-4, and C-reactive protein (CRP) among others have correlated with lung function changes following immunosuppressive treatment in SSc-ILD (51, 169), RA-ILD (170), and IIM-ILD (171). Assassi and colleagues recently developed a prediction model for response to cyclophosphamide and mycophenolate in SSc-ILD using interferon-induced serum proteins (172). Ikeda et al. showed plasma SP-D concentration may predict favorable response to pirfenidone (173) in patients with IPF, though Neighbors and colleagues showed consistent pirfenidone treatment effect irrespective of plasma concentration for a panel of prospectively collected candidate biomarkers (126).

Genomic biomarkers have also suggested potential for predicting differential treatment response. Two *post-hoc* analyses of the PANTHER-IPF trial have been of particular interest. The first showed a common gene polymorphism in *TOLLIP* to predict differential progression in patients treated with *N*-acetylcysteine (174). The next showed short leukocyte telomere length was associated with worse outcome in patients treated with immunosuppressant therapy (175). The former provided the rationale for the recently funded IPF PRECISIONS trial (NCT 04300920) which will test this observation prospectively, while the latter has raised the question of whether immunosuppressant therapy should be used for other forms of ILD in the setting of short telomere length. Adegunsoye et al. recently demonstrated similar outcomes among CHP patients with short telomere length treated with mycophenolate mofetil, though sample size was small (176). At present, telomere length does not appear to influence response to anti-fibrotic therapy in patients with IPF (177).

## Airway Biomarkers

### Diagnostic

Bronchoalveolar lavage (BAL) has a long and controversial history in the evaluation of ILD (178, 179). Its primary use remains discriminating CHP from other forms of ILD, which is supported by elevated lymphocyte count (180). Accordingly, BAL with cellular analysis is now formally recommended to assist in the diagnosis of CHP (181) and has received a conditional recommendation for excluding other potential ILD causes when diagnosing IPF (182). BAL protein biomarkers may help differentiate ILD subtypes, including organizing pneumonia from UIP in patients with RA-ILD (183), IPF from other forms of ILD (93, 184–186), as well as CHP from IPF (100). Exhaled breath analysis of volatile organic compounds is an evolving, but promising modality for discriminating ILD subtypes that may 1 day provide a non-invasive alternative to BAL (187).

### Prognostic

Airway biomarkers of ILD outcomes have been identified, but studies are limited relative to those performed in peripheral blood. Some studies have assessed whether BAL cellular makeup can predict outcome in patients with different ILD subtypes, but results have had mixed (188–191). BAL protein biomarkers, including YKL-40, IL-15, IL-2, and TNF have been linked to differential survival in various ILD subtypes (101, 150, 192), but these studies have been generally limited by small sample sizes. Airway biomarkers of near-term FVC function decline are also limited, but BAL interferon gamma and transforming growth factor beta may predict progressive RA-ILD (193). Norman et al. recently showed that a BAL fluid proteomic signature may predict near-term IPF progression, with high concentrations of immune-regulatory proteins being associated with slower progression (194).

## Parenchymal Lung Biomarkers

### Diagnostic

Transcriptomic analysis of lung tissue has emerged as an exciting arena of biomarker research. Furusawa et al. recently employed this methodology to identify gene signatures unique to patients with IPF and CHP that may allow for better diagnostic discrimination if this can be implemented using less invasive measures (195). Transcriptomic analysis of lung tissue obtained by transbronchial biopsy has resulted in the first commercially available ILD biomarker. The Envisia® genomic classifier (Veracyte, South San Francisco, CA) predicts the presence of histologic usual interstitial pneumonia using a proprietary gene expression-based signature (196). This tool has been shown to predict histologic UIP with good test performance and increases diagnostic confidence for IPF when positive (197, 198).

Several unanswered questions need to be addressed before widespread use of Envisia in the ILD evaluation. First, because UIP can be observed in other ILD subtypes, including RA-ILD, SSc-ILD, asbestosis, and CHP (2, 199–202), it remains unclear how well this molecular diagnostic tool discriminates UIP due to IPF from UIP seen in these other conditions. Next, the radiologic pattern for which this tool is most useful remains unclear. Because those with probable UIP on HRCT can meet IPF criteria in the appropriate clinical setting (203, 204), the utility of genomic UIP in these patients is unclear. Additionally, because those with an alternative diagnosis pattern on HRCT can fail to meet IPF criteria even in the setting of histologic UIP (182), the meaning of genomic UIP in such patients remains unclear. Finally, it remains unclear whether the phenotype identified by this molecular diagnostic tool approximates that of IPF in other ILD subtypes. Demonstrating that genomic UIP follows an IPF-like progressive phenotype will be important to support its clinical utility.

### Prognostic

Few parenchymal lung biomarkers have been identified to predict outcomes in patients with ILD. Telomere length in type II alveolar cells is a notable exception that predicts decreased survival in patients with IPF (205). However, telomere length was recently shown to correlate across human tissues (206), suggesting that telomere length measurement in peripheral blood provides a safer and easier alternative to parenchymal-based measures.

## Unmet Needs in ILD Biomarker Research

While the last two decades have seen impressive progress made in biomarker discovery, a number of unmet needs remain. First, as PF-ILD evolves as a phenotype, the identification and implementation of biomarkers that predict PF-ILD will be critical to our success as a community. Proposed criteria for identifying patients with PF-ILD rely on objective markers of ILD progression to manifest. Predicting progression before it occurs and initiating appropriate therapy remains our best chance to prevent irreversible fibrosis from developing. Biomarkers could also mitigate the uncertainty surrounding prognosis, CT surveillance, and need for early therapy in the substantial number of asymptomatic older adults with incidentally discovered ILAs. Next, while survival is perhaps the most important ILD outcome, biomarkers of survival are less likely to influence near-term treatment decisions or clinical trial design. Because change in FVC is commonly used to assess treatment response, biomarkers predictive of near-term change in FVC are more likely to be informative and therefore implemented. Such biomarkers also have high potential to enrich clinical trial cohorts with patients most likely to experience near-term FVC decline, thereby reducing sample sizes needed to detect treatment effect. Finally, our ability to predict which patients will respond favorably to specific ILD therapies remains limited. Current measures of ILD disease progression may reflect worsening inflammation or progressive fibrosis but cannot differentiate between the two. The means to reliably discriminate inflammatory from fibrotic lung injury would allow us to predict favorable response to immunosuppression or antifibrotic treatments that could halt or potentially reverse loss of lung function. For precision medicine to become a reality, we must have viable biomarkers to guide this approach.

We and others have demonstrated the ability of aggregated biomarkers to augment risk explanation when compared to biomarkers modeled in isolation. Nearly all validated clinical prediction models for predicting ILD outcomes utilize several clinical parameters. A similar approach is likely necessary with biomarker modeling. Multi-dimensional prediction models that incorporate clinical and molecular data may be better yet. With the emergence of high-throughput genomic, transcriptomic, and proteomic platforms, a number of novel and informative ILD biomarkers are likely to emerge over the next decade. A mechanism to quickly and seamlessly incorporate these novel biomarkers into existing prediction tools will be critical for moving the field forward.

## Conclusion

A host of biomarkers drawn from peripheral blood, airway, and parenchymal compartments have proven informative in patients with ILD. These include genomic, transcriptomic, and proteomic determinants of ILD presence, prognosis, and treatment response. While most studies have used IPF as the prototypical PF-ILD, a rapid expansion of this research to include other ILD subtypes meeting validated criteria for PF-ILD is expected in the coming years. Such investigations have high potential to identify biomarkers that transcend clinical ILD diagnosis and instead identify a homogeneous PF-ILD endotype.

## Author Contributions

WB, JO, and GE: literature review. WB and JO: manuscript preparation. All authors contributed to the article and approved the submitted version.

## Conflict of Interest

JO has received consultancy or speaker fees from Boehringer Ingelheim and Genentech, Inc. The remaining authors declare that the research was conducted in the absence of any commercial or financial relationships that could be construed as a potential conflict of interest.
